# Malate dehydrogenase in parasitic protozoans: roles in metabolism and potential therapeutic applications

**DOI:** 10.1042/EBC20230075

**Published:** 2024-10-03

**Authors:** Amy L. Springer, Swati Agrawal, Eric P. Chang

**Affiliations:** 1 Department of Biochemistry and Molecular Biology, University of Massachusetts, Amherst, MA, U.S.A.; 2 Department of Biological Sciences, University of Mary Washington, Fredericksburg, VA, U.S.A.; 3 Department of Chemistry and Physical Sciences, Pace University, New York, NY, U.S.A.

**Keywords:** Apicomplexan, Kinetoplastid, MDH, parasitic protozoa

## Abstract

The role of malate dehydrogenase (MDH) in the metabolism of various medically significant protozoan parasites is reviewed. MDH is an NADH-dependent oxidoreductase that catalyzes interconversion between oxaloacetate and malate, provides metabolic intermediates for both catabolic and anabolic pathways, and can contribute to NAD+/NADH balance in multiple cellular compartments. MDH is present in nearly all organisms; isoforms of MDH from apicomplexans (*Plasmodium falciparum*, *Toxoplasma gondii, Cryptosporidium spp*.), trypanosomatids (*Trypanosoma brucei, T. cruzi*) and anaerobic protozoans (*Trichomonas vaginalis, Giardia duodenalis*) are presented here. Many parasitic species have complex life cycles and depend on the environment of their hosts for carbon sources and other nutrients. Metabolic plasticity is crucial to parasite transition between host environments; thus, the regulation of metabolic processes is an important area to explore for therapeutic intervention. Common themes in protozoan parasite metabolism include emphasis on glycolytic catabolism, substrate-level phosphorylation, non-traditional uses of common pathways like tricarboxylic acid cycle and adapted or reduced mitochondria-like organelles. We describe the roles of MDH isoforms in these pathways, discuss unusual structural or functional features of these isoforms relevant to activity or drug targeting, and review current studies exploring the therapeutic potential of MDH and related genes. These studies show that MDH activity has important roles in many metabolic pathways, and thus in the metabolic transitions of protozoan parasites needed for success as pathogens.

## Introduction

Protozoan parasites are responsible for a variety of diseases, particularly in the developing world where infection of humans and livestock contributes to cycles of poverty [1], so that treatments need to be easy to deliver and low cost. Moreover, because of the risk of acquired resistance, there is always a need for new therapeutic approaches in the pipeline [2,3]. Protozoan parasites are quite taxonomically and metabolically diverse, so development of effective therapies requires a thorough understanding of their biology. Many species depend on the environment of their hosts for carbon sources and other nutrients. The ability to adjust metabolic flux is crucial to parasite survival; thus, regulation of metabolic processes is an important area to explore for therapeutic intervention [4]. In this review, we will focus on a highly conserved metabolic enzyme, malate dehydrogenase (MDH), an NADH-dependent oxidoreductase that catalyzes interconversion between oxaloacetate (OAA) and malate [5]. This enzyme participates in the malate/aspartate shuttle that moves reducing power across the mitochondrial membrane, generating OAA for gluconeogenesis, fatty acid synthesis and amino acid synthesis [5]. In providing substrates for both catabolic and anabolic pathways, MDH contributes to metabolic plasticity.

The parasitic species discussed here often have complex life cycles needed for survival and pathogenesis; different life stages can have distinct patterns of metabolic flux and nutrient utilization. Common themes include emphasis on glycolysis and substrate-level phosphorylation, absence of common pathways like TCA cycle (or use of these enzymes in non-traditional ways), and adapted or reduced mitochondria-like organelles [6,7]. MDH is present in all these species, some of which have multiple MDH isoforms with specific cellular localizations. MDH activity can provide intermediates and reducing equivalents for multiple pathways, and thus has a central role in the success of metabolic transitions between life stages.

### Roles of MDH in various medically significant parasites

To summarize current understanding about roles of MDH in some representative parasitic species, we provide an overview of what is known about the respective MDH isoforms and metabolic pathways that influence flux of intermediates to or from MDH. Although little is yet known about specific contributions of MDH to pathogenicity in parasitic protozoans, our aim is to direct those interested in this field to the relevant literature. Although there is not space in this review for detailed analysis of each MDH isoform, the VEuPathDB (veupathdb.org; [8]) is an excellent resource to access genomic/large-scale databases and analysis tools for infectious disease pathogens, providing current information about MDH annotation, expression patterns or localization.

#### Apicomplexans

The phylum *Apicomplexa* consists of parasitic alveolates responsible for causing devastating diseases in humans and animals, including cryptosporidiosis (caused by *Cryptosporidium spp*.), malaria (*Plasmodium spp*.), toxoplasmosis (*Toxoplasma spp*.), cyclosporiasis (*Cyclospora spp*.), and babesiosis (*Babesia spp*.). They are obligate intracellular parasites with complex life cycles, typically undergoing both asexual and sexual replication cycles. Apicomplexans are characterized by an apical complex structure essential for successful entry and exit out of the host cell [9]. Most apicomplexans contain an unusual non-photosynthetic plastid organelle called an apicoplast [10]. MDH isoforms in *Apicomplexa* species are derived via lateral gene transfer from α-proteobacteria, distinct from the γ-proteobacterial origin of most mitochondrial MDHs [11]. Furthermore, a gene duplication event in *Apicomplexa* led to convergent evolution of lactate dehydrogenase (LDH), which catalyzes the interconversion between pyruvate and lactate. LDH is structurally very similar to MDH but has a distinct substrate specificity [12,13] that is typically determined by a single residue within the active site, the exact residue and position varies depending on the isoform [13,14].

**Figure 1:**
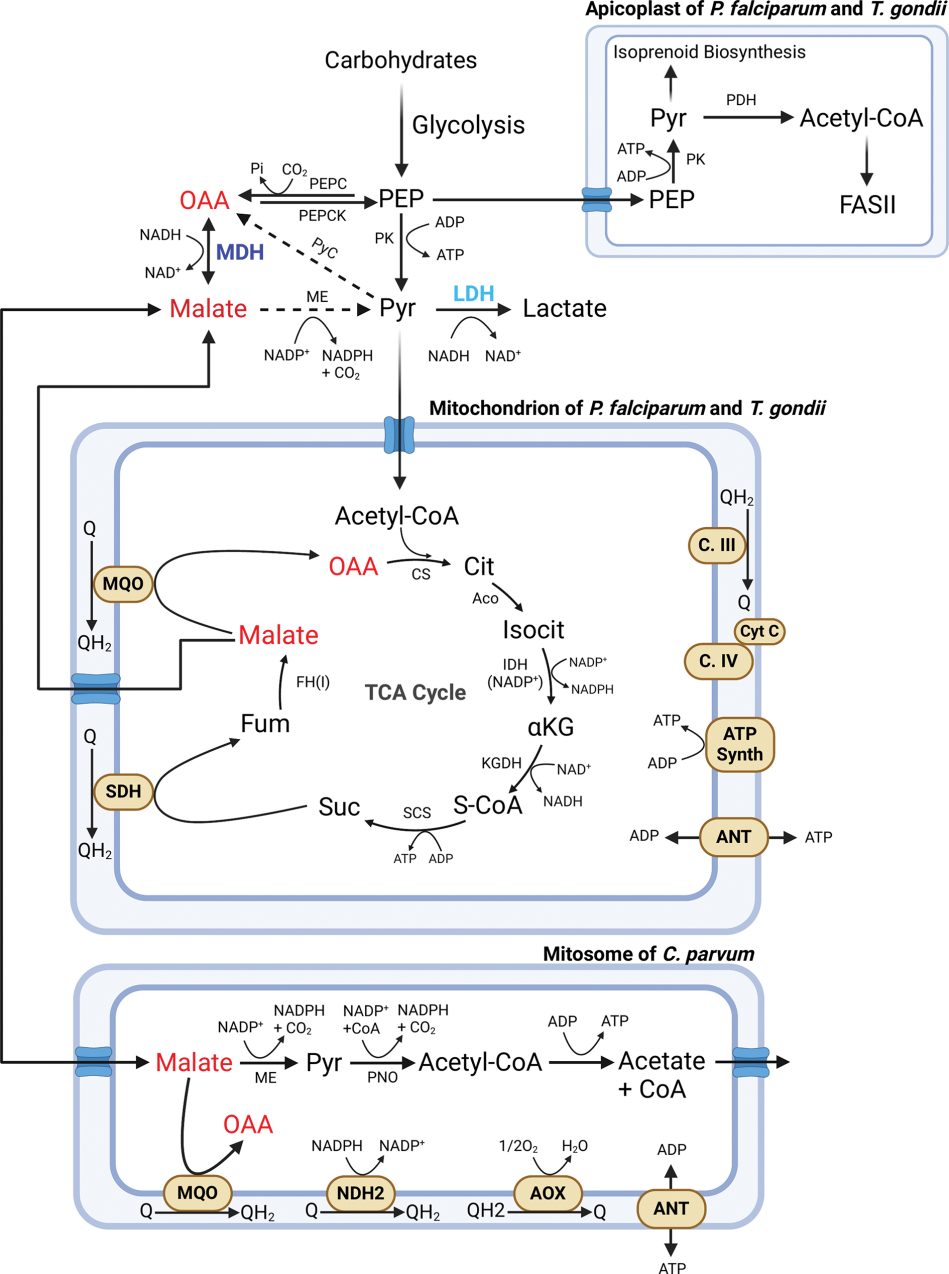
MDH-related metabolism in apicomplexans *P. falciparum*, *T. gondii* and *C. parvum* This schematic highlights key pathways in cytoplasm and some organelles: mitochondria (*P. falciparum* and *T. gondii*), apicoplast (*P. falciparum* and *T. gondii*) and mitosome *(C. parvum*). MDH and LDH are colored purple and blue, respectively. MDH substrates malate and oxaloacetate are indicated in red. Complex I and some other common elements are omitted for clarity. Abbreviations: αKG, α-ketoglutarate; Aco, aconitase; ADP, adenosine diphosphate; ANT, adenine nucleotide translocator; AOX Alternative respiratory oxidase; ATP Synth, ATP synthase; C.III, complex III; C. IV, complex IV; Cit, citrate; CS, citrate synthase; CytC, cytochrome *c*; FASII, fatty acid biosynthesis type II; FH, fumarate hydratase; Fum, fumarate; IDH, isocitrate dehydrogenase; Isocit, isocitrate; KGDH, α-ketoglutarate dehydrogenase; ME, malic enzyme; MQO, malate-quinone oxidoreductase; NDH2, alternative type II NADH dehydrogenase; OAA, oxaloacetate; PDH, pyruvate dehydrogenase; PEP, phosphoenolpyruvate; PEPCK, PEP carboxykinase; Pi, inorganic phosphate; PK, pyruvate kinase; Pyc, pyruvate carboxylase; Pyr, pyruvate; Q/QH2, coenzyme Q oxidized/reduced forms; S-CoA, succinyl-CoA; SCS, succinyl-CoA synthetase; SDH, succinate dehydrogenase; Suc, succinate. For more complete metabolic figures, see [7,41,42,43]. Created with BioRender.com.

*Plasmodium* spp. are the causative agents for malaria. Two species: *Plasmodium falciparum and P. vivax*, are responsible for the majority of cases of human malaria, resulting in more than half a million deaths worldwide every year [15]. The life cycle of these parasites includes sexual development in the mosquito vector, asexual liver-stage, and sexual and asexual stages inside erythrocytes. These organisms show dramatically different metabolic flux profiles in different life stages, suggesting control of metabolic flux is important for survival [16]. During erythrocytic growth, metabolism in *Plasmodium* spp. is predominantly glycolytic [17,18], with ATP generated by substrate-level phosphorylation. *P. falciparum* MDH (PfMDH, Table 1) has been well-characterized structurally and functionally [19–23], in the cytoplasm PfMDH regenerates NAD+ for glycolysis [17,20], supplies OAA for biosynthetic processes [7], and malate for transport into the mitochondria for other catabolism (Figure 1) [24]. *P. falciparum* has a conventional TCA cycle in the mitochondrion except that it lacks MDH and instead uses malate-quinone oxidoreductase (MQO), which irreversibly converts malate to OAA [25]. PfMDH is structurally very similar to PfLDH [13,26], and both have a role in NAD+ regeneration and providing substrates for catabolic and anabolic pathways, although expression patterns differ slightly during erythrocytic growth [27] in that PfMDH levels modulate with growth rate while PfLDH levels remain high regardless of growth state. The potential of LDH and MDH as therapeutic targets has been reviewed elsewhere [28].

**Table 1:** List of MDH and LDH isoforms discussed in this review

Name	Organism^1^	Localization	Key pathways^2^	UniProt ID	Genbank ID^3^	PDB^4^	Reference for Protein sequence
CpLDH	*C. parvum*	Cytoplasm	NadR	Q5CYZ2	AF274310	4ND1	[12]
CpMDH	*C. parvum*	Cytoplasm	NadR, PHR	Q5CYZ3	XM_628235.1	2HJR	[12]
GdMDH	*G duodenalis*	Cytoplasm	NadR, PHR	Q9Y1U1	AF076964	–	[29]
PfLDH	*P. falciparum*	Cytoplasm	NadR	Q27743	M93720.1	2A94	[26]
PfMDH	*P. falciparum*	Cytoplasm	NadR, PHR	Q6VVP7	AY324107	6Y91	[20]
TbcMDH	*T. brucei*	Cytoplasm	MAS, PHR, Gluc	Q95WV4	AF287299.1	–	[30]
TbgMDH	*T. brucei*	Glycosome	NadR	Q387X2	XM_822919.1	–	[31]
TbmMDH	*T. brucei*	Mitochondria	FA	O15769	XM_817416.1	–	[32]
TcAHADH^5^	*T. cruzi*	Cytoplasm	No MDH activity	Q4CTR7	XM_800424.1	–	[33]
TcgMDH	*T. cruzi*	Glycosome	NadR	Q4DRD8	XM_811855.1	7NRZ	[34]
TcmMDH	*T. cruzi*	Mitochondria	MAS	Q4D4A0	AF051893.1	–	[35]
TgMDH	*T. gondii*	Mitochondria^6^	MAS, Gluc	V4Z6Z1	AAX83290	–	[36,37]
TgLDH	*T. gondii*	Cytoplasm	NadR	A0A0F7UY31	LN714498.1	1PZF	[38]
TvLDH	*T. vaginalis*	Cytoplasm	NadR	A2FKC7	XM_001327757.2	–	[39]
TvMDH	*T. vaginalis*	Cytoplasm	PHR, AA	A2DMN2	XM_001579189.2	–	[39]
**Non-parasitic MDHs**							
α-proteo	*Multi-species*	Cytoplasm	TCA cycle	–	WP_204195939.1	–	NCBI
HMDH1	*Homo sapiens*	Cytoplasm	MAS, NadR	P40925	D55654.1	7RM9	NCBI
HMDH2	*Homo sapiens*	Mitochondria	TCA cycle	P40926	AF047470.1	2DFD	NCBI

1 For current genomic and proteomic information on parasitic MDHs, refer to https://veupathdb.org [8].

2 Abbreviations for Pathways: NadR = NAD+ regeneration (for glycolysis), MAS = Mal/Asp shuttle, FA = Fatty acid synthesis, AA = amino acid catabolism, PHR = NADPH regeneration, Gluc = gluconeogenesis. Not all pathways are listed, only a few key examples.

3 Introns are rare in Trypanosoma, Trichomonas and Giardia, and none are reported in the MDH and LDH genes reported here.

4 PDB code is reported when a structure is available.

5 The gene is annotated in genome reports as cMDH, however leading to some confusion in the literature.

6 See note in text about localization [40].

Structurally, PfLDH has a feature distinct from PfMDH, a 5 amino acid loop that protrudes from a portion of the substrate binding pocket [44]. Apicomplexan PfLDH and PfMDH lack one residue in the highly conserved N-terminal NADH binding region (GX-GXXG instead of GXXGXXG) relative to MDH isoforms from other organisms [20,21]. Although small, these differences in binding pocket features such as these may be informative for design of competitive inhibitors. Since there is some redundancy of function between PfMDH and PfLDH, dual inhibitors that inactivate both PfMDH and PfLDH may be necessary for effective therapy [20].

Another medically significant apicomplexan parasite is *Toxoplasma gondii*, known to infect almost one-third of the global human population [45], due in part to its ability to invade nearly any type of mammalian cell [46]. Although many infections are largely asymptomatic, infection can be fatal in immunocompromised individuals [45]. ATP production is largely glycolytic [47], but a functional TCA cycle, mitochondrial electron transport chain and gluconeogenesis are also required [48]. *T. gondii* expresses MDH (TgMDH, Table 1 [37]) in all developmental stages. TgMDH was reported to localize to mitochondria [36]; however, spatial proteome mapping shows localization in cytoplasm [40] suggesting further investigation is required [18]. *T. gondii* has mitochondrial MQO [49], so if TgMDH is mitochondrial there is redundancy of function. If in the cytoplasm, TgMDH might have similar roles to those described above for PfMDH (Figure 1), as well as provide OAA for gluconeogenesis [48]. TgMDH was not found to be essential for fitness in the rapidly growing tachyzoite stage *in vitro* [50], yet it may be important for growth in different hosts, when ability to adapt to changes in nutrient availability is critical.

*Cryptosporidium* spp. are parasites that infect intestinal enterocytes in humans and animals [51], typically spread via contaminated water supplies [52]. Cryptosporidiosis is a diarrhoeal disease that is non-fatal to healthy individuals but can be deadly in young children and the immunocompromised [53]. *Cryptosporidium parvum* and *C. hominis* are the most virulent species, making up the majority of human cases [53]. *Cryptosporidium* spp. are unusual among the *Apicomplexa* as they lack apicoplasts [54] and have rudimentary mitochondria known as mitosomes [55,56], an organelle whose complexity and components vary among *Cryptosporidium* spp [56]. Oocysts can survive for long periods in contaminated water [57], but upon invasion of the host, have a single-host life cycle that alternates between asexual and sexual reproduction [58]. *C. parvum* has limited biosynthetic capacity and is completely dependent on its host to obtain key nutrients [58,59].

*C. parvum* has a cytoplasmic MDH (CpMDH, Table 1) important for regeneration of NAD+ and for providing malate for malic enzyme (ME) catalysis, reducing NADP+ to NADPH (Figure 1) [41,42]. CpLDH, evolved from CpMDH through a gene duplication event that occurred after *C. parvum* diverged from other apicomplexans [12], also plays a major role in NAD+ regeneration [60]. *C. parvum* mitosomes lack most TCA cycle enzymes but have MQO [43, 61, 62] to generate OAA for synthesis of citrate for biosynthetic pathways (Figure 1). Another role proposed for CpMDH is to provide malate for mitosomes, where mitosomal ME converts it to pyruvate for substrate-level phosphorylation [43]. In these roles, CpMDH redirects high metabolic flux through regeneration of NAD+ or NADPH and contributes to ATP production as per the energetic needs of the cell.

#### Trypanosomatids

Trypanosomatids are flagellated protozoans in the class *Kinetoplastea*, and are characterized by a large single flagellum, a cytoskeletal array of sub-pellicular microtubules, and an unusual DNA-containing structure in their mitochondrion called a kinetoplast [63,64]. Trypanosomatid species are all pathogenic and typically have a complex life cycle involving multiple hosts such as an insect vector and a vertebrate host [65–67]. These organisms carry out many basic biochemical processes, but have some unusual features including a specialized peroxisomal organelle, the glycosome, which compartmentalizes the first seven reactions of glycolysis, among other pathways [67–69]. ATP is generated primarily by substrate-level phosphorylation and fermentation to regenerate reducing power [65,67]. Pathogen survival requires successful differentiation between life stages [4,70], and MDH isoforms are central to the metabolic processes that support these transitions.

*Trypanosoma brucei* has life stages as an extracellular parasite in the tsetse fly and the bloodstream of mammals including humans [66,71], where it causes the central nervous system disease African sleeping sickness, that is fatal if not treated. The disease also impacts livestock populations in sub-saharan Africa [72]. In the insect stage, *T. brucei* procyclic trypomastigote grows oxidatively using amino acids, particularly proline, as carbon sources [68,73]. ATP generation occurs from substrate-level phosphorylation in the glycosome and the cytoplasm [65,74]. At this stage, the cell has a single large mitochondrion that expresses TCA cycle enzymes; however, the primary source of ATP production is not through a canonical TCA cycle. Instead these enzymes are probably used primarily for biosynthetic pathways [74–76]. Upon transition to the bloodstream form, metacyclic trypomastigote, the mitochondrion is smaller, lacks cristae, and many metabolic enzymes are down regulated [71,77,78]. In this stage, energy metabolism is primarily from glycolysis, with ATP generated from substrate-level phosphorylation as pyruvate is produced in the cytoplasm [76,79].

**Figure 2:**
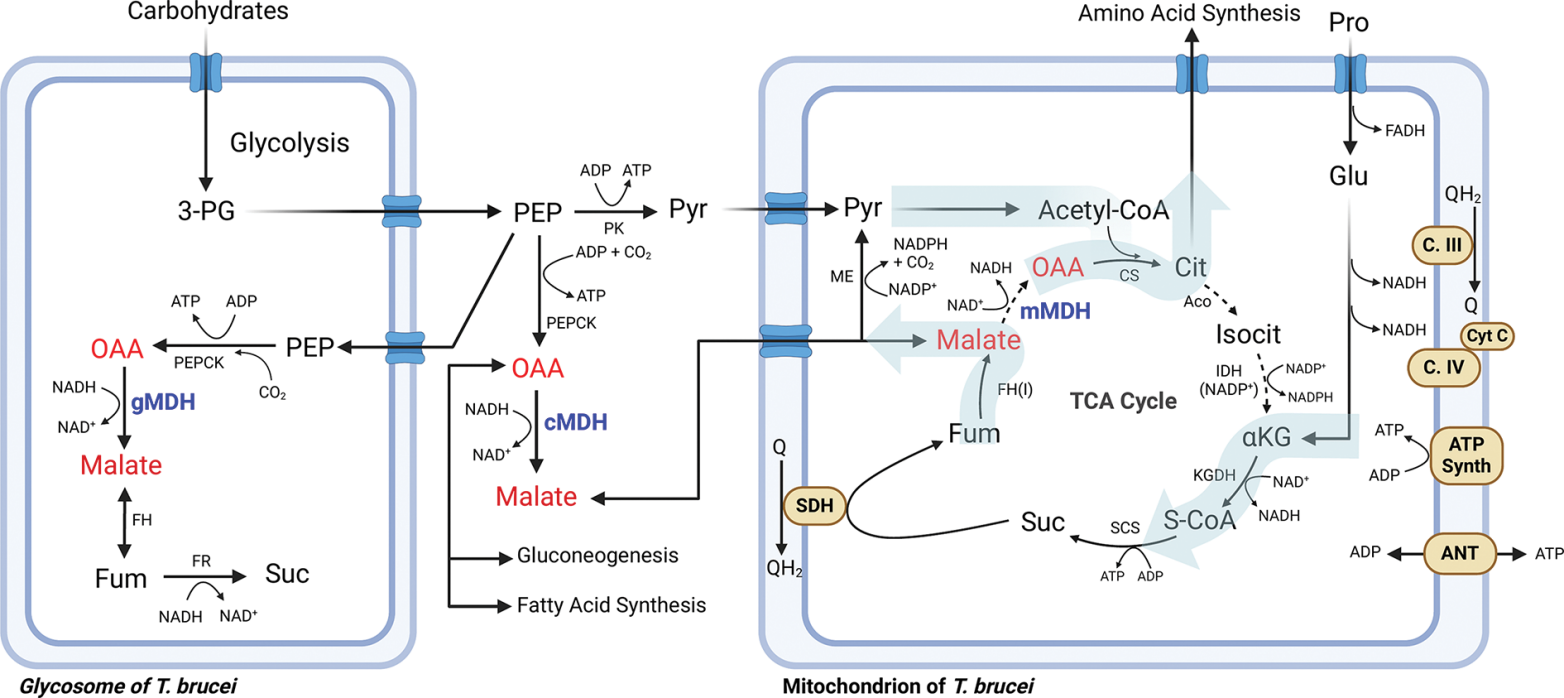
MDH-related metabolism in *T. brucei* Highlighting key pathways in cytoplasm, mitochondrion and glycosome. MDH is colored purple, MDH substrates malate and oxaloacetate are indicated in red. Colored thick arrows over the TCA cycle represent functions of those enzymes in the insect stage, adapted from [75]. Complex I and some other common elements are omitted for clarity. Abbreviations: αKG, α-ketoglutarate; 3-PG, 3-phosphoglycerate; Aco, aconitase; ADP, adenosine diphosphate; ANT, adenine nucleotide translocator; ATP Synth, ATP synthase; C.III, complex III; C. IV, complex IV; Cit, citrate; CS, citrate synthase; CytC, cytochrome *c*; FADH, flavin adenine dinucleotide (reduced); FH, fumarate hydratase; FR, NADH-dependent fumarate reductase; Fum, fumarate; Glu, glutamate; IDH, isocitrate dehydrogenase; Isocit, isocitrate; KGDH, α-ketoglutarate dehydrogenase; ME, malic enzyme; OAA, oxaloacetate; PEP, phosphoenolpyruvate; PEPCK, PEP carboxykinase; PK, pyruvate kinase; Pro, proline; Pyr, pyruvate; Q/QH2, coenzyme Q oxidized/reduced forms; S-CoA, succinyl-CoA; SCS, succinyl-CoA synthetase; SDH, succinate dehydrogenase; Suc, succinate. For more complete metabolic figures, see [75]. Created with BioRender.com.

*T. brucei* has three isoforms of MDH (Table 1), localized to cytoplasm (TbcMDH), mitochondria (TbmMDH) and glycosome (TbgMDH), respectively [30–32, 80, 81], shown in Figure 2. Unlike in *Apicomplexa*, trypanosome MDHs are derived from eukaryotic cytoplasmic and mitochondrial lineages [12,81,33]. Notably, *T. brucei*, *T. cruzi* and *Leishmania* species do not contain LDH genes [67,82]. All three MDH isoforms are expressed in the insect stage, during which TbmMDH has the greatest activity [81]. In the bloodstream form, only TbcMDH activity is detected [30,81], although TbmMDH and TbgMDH may be present at low levels. TbgMDH is important in insect stage for regenerating NAD+ within the glycosome for glycolytic reactions, for generating malate that is exported to be a ME substrate, and provide OAA for gluconeogenesis [69,74]. TbcMDH is expressed in all life stages, where it is likely participating in the malate/aspartate shuttle to maintain NAD+/NADH balance between cytoplasmic and mitochondrial compartments and gluconeogenesis [75,76,81]. TbmMDH, expressed in the insect stage, can participate in TCA cycle [83] but predominantly generates malate for export for cytoplasmic fatty acid synthesis and NADPH regeneration [75,76]. Although mMDH activity is not detected in the bloodstream form, proteomic analysis shows TbmMDH up-regulated during transition from the insect stage to the bloodstream form [84], suggesting some roles of MDH have yet to be described.

Another medically significant *Trypanosoma* species, *T. cruzi* [85,86] has an insect stage (epimastigote), a mammalian-infective bloodstream stage (metacyclic trypomastigote) and an intracellular phase in mammalian cells (amastigote). Metabolic changes are critical for transitions between life stages [87–89]. *T. cruzi* has mitochondrial (TcmMDH) and glycosomal (TcgMDH) isoforms (Table 1 [35, 90, 91]. A third MDH-like gene exists and is annotated as cytoplasmic MDH; however, the encoded protein has no MDH activity and instead is an aromatic alpha-hydroxyacid dehydrogenase (AHADH) [33]. TcmMDH is active in all life stages of *T. cruzi* while TcgMDH is active only in insect stage [92]. TcgMDH is the only trypanosome MDH so far for which a structure is reported [34] which reveals a proline-rich loop of 9 amino acids near an NAD+ binding region that is not found in mammalian MDHs. Features like these that distinguish trypanosome glycosomal MDHs from host MDHs, are appealing as potential therapeutic targets [93]. Other trypanosomatids, such as *Leishmania* species [94], also contain three isoforms of MDH [95,96]. Many recent studies exploring metabolic dynamics in these species [69,83,97,98] highlight the advancement in understanding the essential biological functions in these parasites. Because of the multiple cellular locations, and the variety of pathways that MDH can participate in, MDHs in trypanosomatids are critical for supporting required metabolic transitions, so elucidating the function and regulation of MDH isoforms can accelerate therapeutic strategies.

#### Anaerobic parasitic protozoans

**Figure 3:**
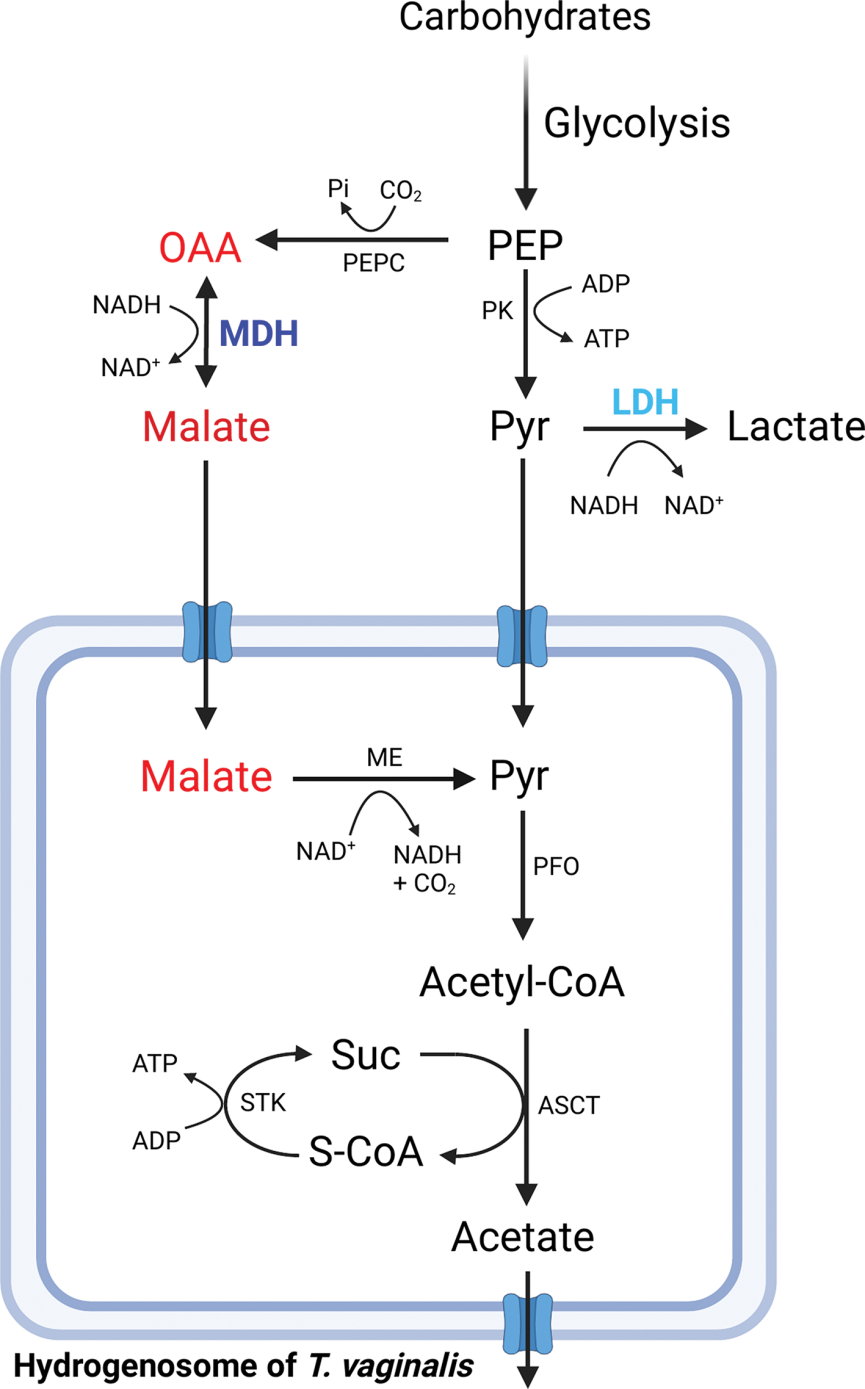
MDH-related metabolism in *T. vaginalis* MDH-related metabolism in *T. vaginalis*, highlighting key pathways in cytoplasm and hydrogenosome. MDH and LDH are colored purple and blue, respectively. MDH substrates malate and oxaloacetate are indicated in red. Complex I and some other common elements are omitted for simplicity. Abbreviations: ASCT, acetate: succinate CoA transferase; ME, malic enzyme; OAA, oxaloacetate; PDH pyruvate dehydrogenase; PEP, phosphoenolpyruvate; PEPC, PEP carboxylase; Pyr, pyruvate; S-CoA, succinyl-CoA; Suc, succinate. For more complete metabolic figures, see [105]. Created with BioRender.com.

The anaerobic parasite protozoans, sometimes referred to as ‘amitochondriate,’ are distinctive in that they are eukaryotes that lack mitochondria and derive energy primarily from anaerobic fermentation. These organisms vary in their core energy metabolism and in metabolic compartmentalization [6]. Two species, *Trichomonas vaginalis*. and *Giardia duodenalis* are described here as examples. *T. vaginalis* is a flagellated protozoan that causes trichomoniasis, a sexually transmitted infection estimated to affect over 1 million individuals in the US each year [99]. Infection can lead to adverse sexual and reproductive health outcomes in both women and men and an increased risk of acquiring HIV and certain types of cancer [100]. *T. vaginalis* uses anaerobic fermentation [101,102] to produce ATP by substrate-level phosphorylation during glycolysis and from further metabolism of pyruvate inside hydrogenosomes, double-membrane enclosed organelles [101] which may have a common eubacterial ancestor with mitochondria [103]. Hydrogenosomes do not contain TCA cycle enzymes but do oxidize pyruvate to yield end products of ATP, acetate, CO_2_ and hydrogen [104]. Pyruvate is either produced from glycolysis, or from the decarboxylation of malate by ME, as shown in Figure 3 [101,105]. *T. vaginalis* has one primary MDH (TvMDH, Table 1), a cytoplasmic isoform of eukaryotic origin [11,13], which generates malate from catabolism of amino acids. TvMDH may be associated peripherally with the hydrogenosome [104], consistent with its role in generating malate for transport into this organelle [106,107]. TvMDH is reported to be down-regulated during glucose restriction, potentially mimicking the transition as infection is established [108]; this regulation may be through and miRNA control of TvMDH [109]. Interestingly, LDH in *T. vaginalis* (TvLDH) is closely related to TvMDH and seems to have arisen from a gene duplication event similar to, but independently of, that proposed for apicomplexan LDH [39]. Another protozoan parasite, *Giardia duodenalis* (also called *G. lamblia* or *G. intestinalis*), is a medically significant intestinal parasite, taxonomically distinct from trichomonads. *G. duodenalis* carries out substrate-level phosphorylation by anaerobic fermentation [110] and lacks conventional mitochondria [103,110] but does contain reduced mitosome compartments, which do not seem to be directly involved in ATP production [6,56,111]. The cytoplasmic MDH (GdMDH, Table 1) is phylogenetically related to that of *T. vaginalis* [29] and predicted to have similar roles (Figure 3) [107], except that malate is metabolized in the cytoplasm. Recent efforts to develop robust genetic manipulation techniques tailored for the double diploid *Giardia* genome will be useful to allow detailed genetic and biochemical characterization of GdMDH [111,112].

In all these parasitic species, MDH is important for catabolism and an appealing therapeutic target [28]. The reliance on glycolytic growth seen in all of these parasites is in some ways reminiscent of the changes to metabolism observed in most types of cancer cells, also known as the ‘Warburg effect’ [113]. A better understanding of the metabolism in these protozoans may provide insight into disease-altered metabolism in mammalian cells as well.

### Structural comparisons of parasitic and mammalian MDHs

**Figure 4:**
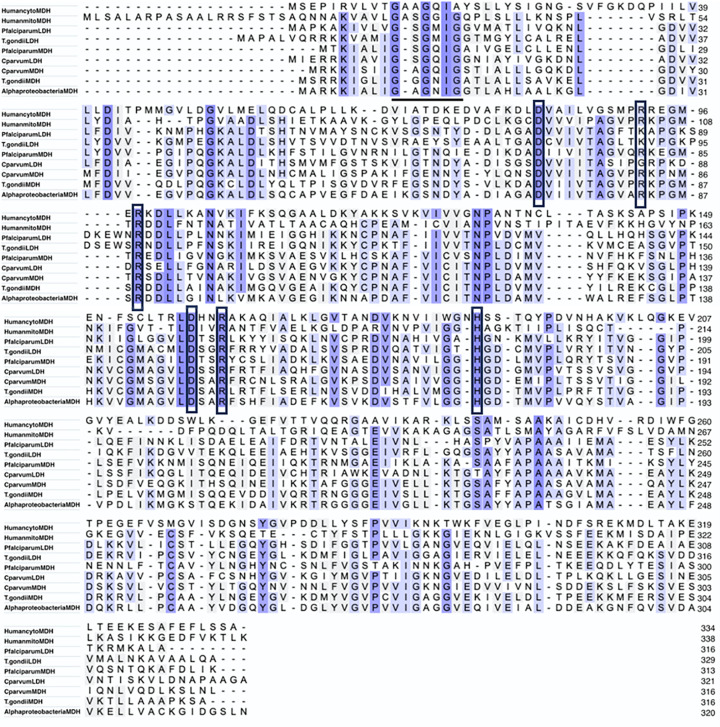
Alignment of Apicomplexan, Human and α-proteobacteria MDH and LDH sequences Sequences used are referenced in Table 1, alignment was performed through UniProt (www.uniprot.org) utilizing the Clustal Omega algorithm (www.clustal.org). Residues important for catalysis and cofactor binding are highlighted in boxes [5]. Gaps have been introduced to produce an optimal alignment. Conserved active site residues are highlighted in boxes. The N-terminal glycine motif which forms the dinucleotide-binding fold in the NADH-binding pocket is underlined, showing the (GXXGXXG) motif, in which apicomplexan species lack one residue. Note that the penultimate residue is isoleucine in each sequence shown in this review.

**Figure 5:**
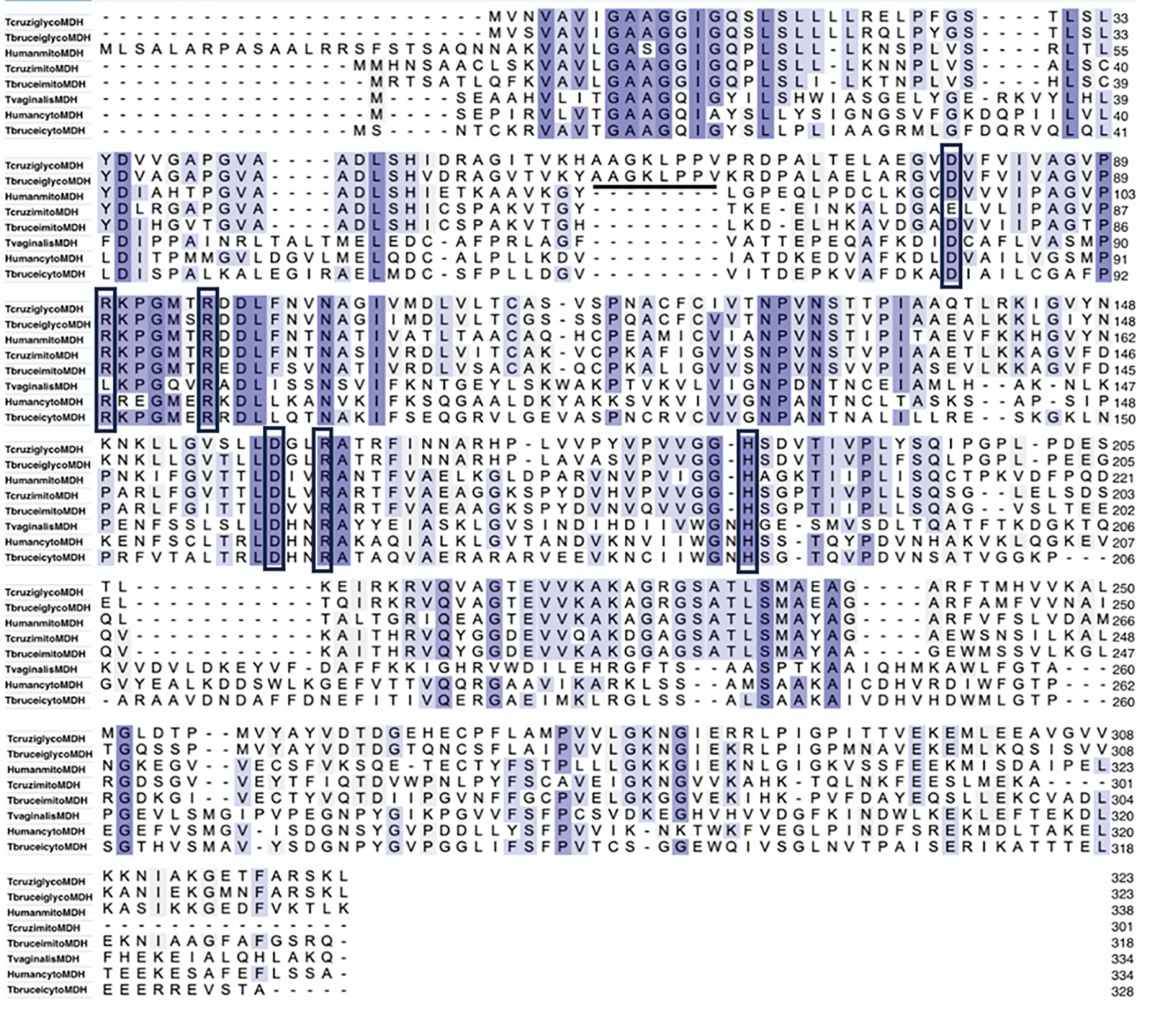
Alignment of trypanosomatid and Human MDH sequences (various isoforms) Alignment of *T. brucei* or *T. cruzi* MDH amino acid sequences with human and α-proteobacteria sequences (Table 1), generated using same procedure as in Figure 4. Where appropriate the MDH isoforms are marked as mitochondrial (mito), glycosomal (glyco) or cytoplasmic (cyto). Conserved residues are shown in boxes and the 9-amino acid proline rich loop in glycosomal MDH is underlined.

**Figure 6:**
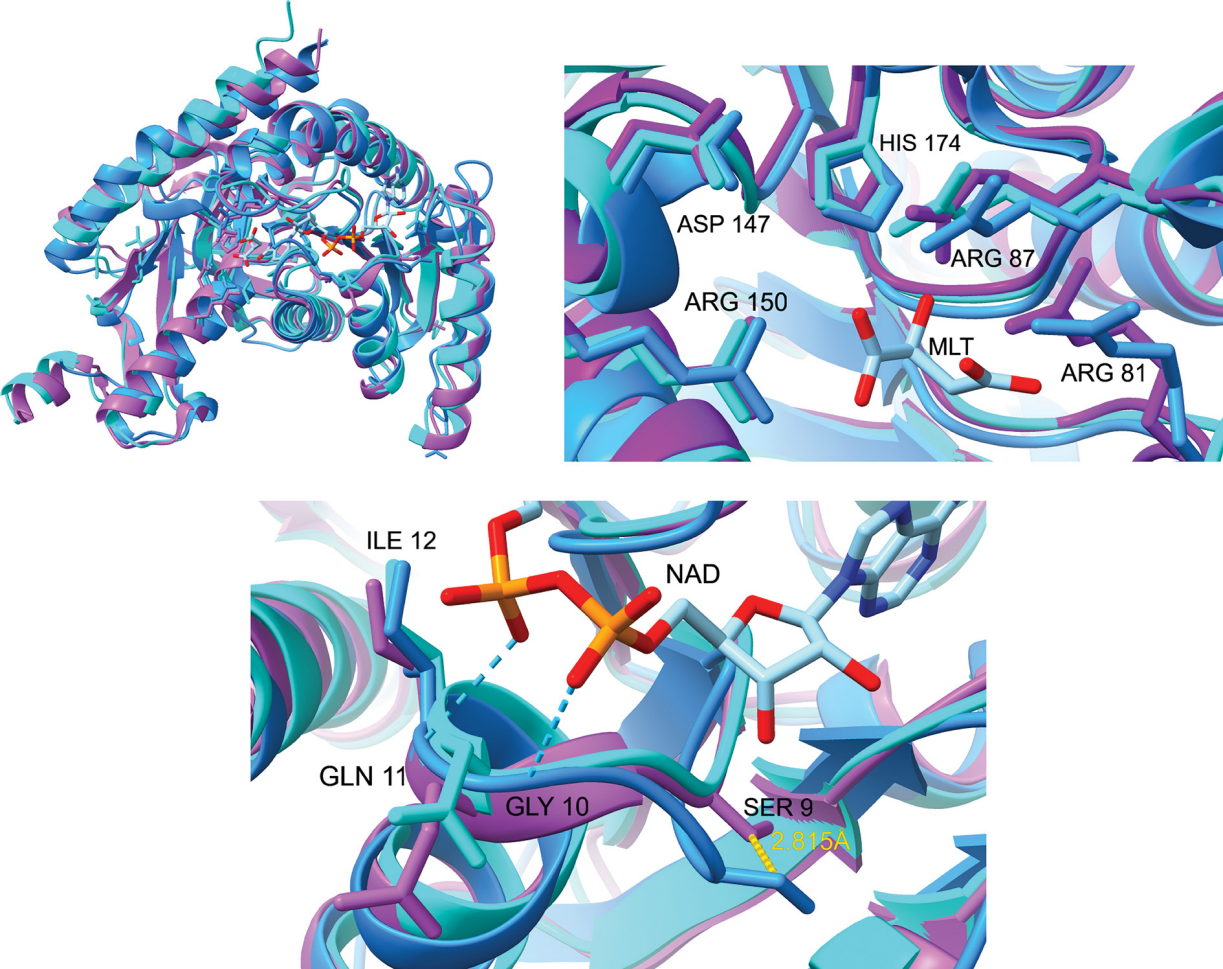
3D rendering of parasitic MDHs and LDHs aligned to Human HMDH2 (Top left) Tertiary structure renderings and alignments performed using ChimeraX Matchmaker tool [114,115] comparing human mitochondrial HMDH2 (blue, PDB code 2DFD), PfMDH (purple, 5NFR), and CpLDH (cyan, 4ND1). Malate and NADH within these renderings are bound to HMDH2. Note the high degree of overlap throughout the structure with the exception of loop and turn regions. (Top Right) Close-up of the active site with malate bound to HMDH2 showing the conservation and alignment of catalytic arginine, histidine and aspartate residues among all three structures, with the exception of CpLDH at position 81, which is glycine 102 in CpLDH sequence. The numbering provided is based on the PfMDH sequence. (Bottom) Close-up NAD binding motif showing hydrogen bonding between the NAD diphosphate oxygens and HMDH2 backbone amide bonds, and the conserved isoleucine. The extra residue in this region causes the HMDH2 loop to bulge out, as shown by the ∼3 angstroms gap between aligned serine residues between PfMDH (Ser 9) and HMDH2.

MDH is found in nearly all organisms, and while the overall structures are conserved, including active site and substrate binding regions, primary amino acid identities between MDH isoforms can be as low as 20% [5,12,29]. To better understand differences between parasitic MDHs and those of their human host, we provide example primary sequence alignments and structural comparisons (Figures 4-6). Apicomplexan MDHs resemble alpha-proteobacterial MDHs (Figure 4), containing all the key catalytic residues, but with some unusual characteristics such as the N-terminal glycine motif noted above [21]. MDHs of other protozoan parasites, such as *T. brucei, T cruzi and T. vaginalis*, derive from eukaryotic mitochondrial and cytoplasmic MDH lineages [12,81]. Primary sequence alignment of *T. brucei, T cruzi, T. vaginalis* and human MDH isoforms (Figure 5) show conservation of catalytic residues, as well as regions conserved among mitochondrial and glycosomal MDH isoforms but not with cytoplasmic MDHs. The distinctive N-terminal 9-amino acid proline-rich loop (AAGPKLPPVP/K) of glycosomal *T. cruzi* and *T. brucei* isoforms is also shown [34]. MDH functions as a homodimer, and higher-order oligomers may exist [12]. Three-dimensional structural alignment of MDHs and LDHs exhibit similar functional structures (Figure 6), [114,115]. Table 2 describes the relatedness of these structures through alignment scores and root-mean-squared deviation RMSD values. Apicomplexan MDH and LDH have higher structural alignment scores and lower RMSD values when compared with human and trypanosomatid MDH isoforms (Table 2), and trypanosomatid TcgMDH shows higher structural alignment with human mitochondrial isoform (HMDH2) than cytoplasmic (HMDH1). A conserved isoleucine residue (PfMDH Ile 12) in the N-terminal glycine motif is aligned in both primary and tertiary structures of apicomplexan and human MDHs while the missing residue in the glycine motif in apicomplexans eliminates a bulge seen in HMDH2 (Figures 4 and 6), which may explain alterations in substrate specificity [21]. Point mutations in this region alter kinetic parameters, typically by increasing *K*_M_, decreasing *k*_cat_, and subsequently lowering enzyme efficiency [21], suggesting structural differences in this region by exploited for drug discovery.

**Table 2:** Comparison of ChimeraX Matchmaker 3D structural alignment scores, atom pairs, and RMSD values for parasitic and human MDH isoforms^*^

Reference structure	Aligned structure	PDB Code	Chain aligned	Alignment score	Pruned atom pairs	Pruned RMSD (Å)	All atom pairs	All RMSD (Å)
**A. Alignment using PfMDH as reference structure**								
PfMDH	PfMDH	5NFR	A	1656.2	313	0	313	0
5NFR	PfMDH	6Y91	B	1597	302	0.407	302	0.407
A chain	CpMDH	2HJR	K	928.4	300	0.807	309	1.015
	CpLDH	4ND1	B	896.9	286	0.954	312	1.411
	TgLDH	1PZF	A	882.4	279	0.887	313	1.455
	PfLDH	2A94	A	880.5	276	0.944	307	1.653
	HMDH1	7RM9	A	459.2	209	1.153	304	2.804
	TcgMDH	7NRZ	G	451.9	164	1.158	287	3.185
	HMDH2	2DFD	A	503.7	188	1.18	295	4.006
**B. Alignment using human HMDH2 as reference structure**								
HMDH2	HMDH2	2DFD	A	1628.8	313	0	313	0
2DFD	TcgMDH	7NRZ	G	920	274	0.785	309	1.726
A chain	CpMDH	2HJR	K	551.2	211	1.07	292	2.391
	CpLDH	4ND1	B	534.5	209	1.167	295	2.552
	TgLDH	1PZF	A	531.9	198	1.149	297	2.615
	PfLDH	2A94	A	469.2	186	1.259	289	2.805
	HMDH1	7RM9	A	434.2	181	1.179	304	3.051
	PfMDH	6Y91	B	495.1	181	1.31	283	3.337
	PfMDH	5NFR	A	503.7	188	1.18	295	4.0006

* The ChimeraX Matchmaker 3D structural alignment tool provides visual (Figure 6) and quantitative information regarding the similarity of tertiary structures. The tables above show the quantitative data output of the alignment scores, atom pairs, and RMSD values using PfMDH and human mitochondrial MDH (HMDH2) as reference structures against the same set of parasitic and mammalian MDH and LDH structures. (**A** and **B**) Matching of PfMDH and HMDH2 structure with itself produced the highest alignment score ranging from 1628-1656, the highest pruned and all-atom pair values of 313, and the lowest RMSD values of zero. Both the pruned and all atom pair values represent the alignment of the c-α carbons between two structures, with pruned pairs having stricter alignment parameters and thus representing a higher degree of structural similarity, typically for a smaller subset of residues. The data are sorted by increasing all atom pairs RMSD values and the alignments were performed using default parameters, i.e., bb chain pairing, Needleman-Wunsch alignment algorithm, BOLUSM-62 similarity matrix, 0.3 SS fraction, 18/18/6 gap open, 1 gap extended, 2 iteration cutoff, and SS matrix HH 6, HS -9, HO -6, SS 6, SO -6, OO 4. (A) The 3D alignment with PfMDH as the reference shows that apicomplexan MDH and LDHs are structurally similar to one another, i.e., higher alignment scores, lower RMSD values, and a higher number of atom pairs. Of the structures sampled, the lowest degree of structural similarity relative to PfMDH were the two mammalian MDH isoforms and *T. cruz*i glycosomal MDH (TcgMDH), i.e., lower alignment scores, fewer atom pairs, and higher RMSD values. For reference, the amino acid identity between PfMDH and HMDH2 is 27.5%, between PfMDH and TcgMDH is 27.4%, and between PfMDH and CpMDH is 43.5%. Note that while the number of pruned atom pairs decreases by 149 and 125 for TcgMDH and HMDH2, respectively, the pruned atom RMSD values remain relatively low, showing that all MDH structures retain a core of well-aligned residues that likely include many in the catalytic site and other conserved structural features. (B) The 3D alignment with HMDH2 as the reference structure shows that HMDH2 does not align as well with the apicomplexan structures or the cytosolic human MDH isoform (HMDH1). Note that the highest degree of similarity to HMDH2 was observed for TcgMDH, suggesting that the two isoforms are evolutionary more related to one another than the other sampled structures. For reference the amino acid identity between TcgMDH and HMD2 is 49.2%. The higher RMSD and lower atom pair values for the HMDH2 alignment suggest that there may be several structural differences that can be selectively targeted in apicomplexan MDHs and LDHs in drug discovery studies. The alignment using HMDH1 as the reference structure (data not shown) produced the lowest overall alignment value, i.e., alignment scores below 500 and all atom pair RMSD values ranging from 2.8 to 4.0 Å for all sampled structures.

### Therapeutic potential and drug discovery of parasitic MDHs

Because these protozoan parasites use non-traditional pathways and unusual organelles, their metabolic enzymes are important for survival and pathogenesis [4]. MDH participates in many of these pathways, making it an attractive target for therapeutic interventions [28]. Therapeutic targeting MDH in specific compartments will need to take into account mechanisms for directing drugs into those compartment or alternatively target the localization of these MDHs [4,116]. While metabolic and structural studies of MDH and related enzymes have been reported for apicomplexan and trypanosomatid parasites, MDH drug discovery remains largely unexplored, and the studies that are published focus mainly on apicomplexans. As discussed above, high structural similarity exists between apicomplexan LDHs and MDHs, so it is possible that some known inhibitors of apicomplexan LDHs might also inhibit apicomplexan MDHs. Furthermore because these proteins have overlapping metabolic roles, dual LDH-MDH inhibitors may have greater efficacy against apicomplexans than inhibitors targeting only one of these enzymes [21,28]. There is a wealth of literature available on apicomplexan LDHs, and this knowledge can inform studies on apicomplexan MDH. Some notable examples include those concerning metabolic roles [117–119], structural properties [14,60,38, 120, 121], and targeted inhibition studies [28,122–129]. Both PfLDH and PfMDH are inhibited by Gossypol, which does not inhibit the corresponding mammalian isoforms [20]. Oxamate, a well-characterized competitive inhibitor of PfLDH, does not show activity against PfMDH; however, some oxamate derivatives inhibit PfMDH at micromolar levels [130]. PfMDH and PfLDH have distinctly different substrate specificity so most studies on targeted inhibition and drug discovery of PfMDH focus on regions distinct from the binding pocket, such as the dimer interface [131]. Certain PfMDH mutations in residues in this region destabilized this interface, resulting in significantly less thermal stability and enzymatic activity. Other mutants that stabilized the interface had significantly higher thermal stability and hypercatalytic activity, and PfMDH heterodimers consisting of one wild-type subunit and one subunit containing a destabilizing mutation showed reduced catalytic efficiency and stability relative to wild-type. These studies demonstrate the potential application of mutations for drug target validation and the significance of PfMDH dimerization for anti-malarial drug discovery [131]. An allosteric pocket for regulating PfMDH has been identified at the dimer interface surface using NMR screening of a fragment library. The fragment 4-(3,4-difluorophenyl) thiazol-2-amine (4DT), and its derivatives, caused structural changes at the active site [132]. A cryptic allosteric site, identified by mutational analysis, affects NADH binding of PfMDH and lies in a region not conserved in human MDH isoforms [133]. Subsequent computational analyses and docking studies involving a panel of ligands were used to identify potential interactions and define surface properties of the site [134]. Studies such as these offer novel avenues for anti-parasitic therapy with minimized risk of off-target effects on human metabolism.

Because MDH isoforms exist in all of these medically significant protozoan parasites, and MDH activity is important for flux through various catabolic and anabolic pathways in each organism, more studies on the function, regulation and structural features of these proteins provide potential for development of effective therapeutic strategies against these pathogens.

## Summary

- A common theme in parasitic energy metabolism is the use of glycolysis and other pathways for substrate-level phosphorylation rather than TCA Cycle, with MDH contributing to NAD+/NADH balance and supplying metabolites for substrate-level phosphorylation.
- Apicomplexan parasites have cytoplasmic MDH but often lack mitochondrial MDH and use a different oxidoreductase (MQO) to convert malate to oxaloacetate in the TCA cycle. Amitochondriate parasites utilize anaerobic fermentation and distinctive metabolic compartmentalization.
- Apicomplexan and amitochondriate parasites have LDH genes that have only recently evolved by gene duplication from MDH, and these enzymes may have overlapping functions in maintaining redox equivalents.
- Trypanosomatids have three isoforms of MDH: cytoplasmic and mitochondrial and glycosomal but lack LDH. They do not have a canonical TCA cycle in mitochondria. The glycosomal MDH maintains NAD+ for glycolysis.
- The unusual metabolic and structural features of parasitic MDHs make them potentially useful as drug targets, and similarities with LDH suggest that non-competitive dual MDH-LDH inhibitors might have greater efficacy than inhibiting either enzyme alone.

## Abbreviations

4DT: 4-(3,4-difluorophenyl) thiazol-2-amine
ATP: adenosine tri-phosphate
*C. hominis*: *Cryptosporidium hominis*
*C. parvum*: *Cryptosporidium parvum*
CpMDH: *C. parvum* MDH
*G. duodenalis*: *Giardia duodenalis*
GdMDH: *G. duodenalis* MDH
*K*_cat_: enzyme catalytic constant
*K*_M_: Michaelis constant, enzyme kinetics
LDH: lactate dehydrogenase
MDH: malate dehydrogenase
ME: malic enzyme
MQO: malate-quinone oxidoreductase
NAD+/NADH: nicotinamide adenine dinucleotide
NADP+/NADPH: nicotinamide adenine dinucleotide phosphate
OAA: oxaloacetate
*P. falciparum*: *Plasmodium falciparum*
PfLDH: *P. falciparum* LDH
PfMDH: *P. falciparum* MDH
RMSD: Root mean square deviation
*T vaginalis*: *Trichomonas vaginalis*
*T. brucei*: *Trypanosoma brucei*
*T. cruzi*: *Trypanosoma cruzi*
*T. gondii*: *Toxoplasma gondii*
TbcMDH: *T. brucei* cytoplasmic MDH
TbgMDH: *T. brucei* glycosomal MDH
TbmMDH: *T. brucei* mitochondrial MDH
TCA cycle: Tricarboxylic acid cycle
TcAHADH *T. cruzi*: aromatic alpha-hydroxyacid dehydrogenase
TcgMDH: *T. cruzi* glycosomal MDH
TcmMDH: *T. cruzi* mitochondrial MDH
TgMDH: *T. gondii* MDH
TvMDH: *T. vaginalis* MDH
